# Editorial: Emergency and critical care of severely injured patients

**DOI:** 10.3389/fmed.2024.1453497

**Published:** 2024-07-15

**Authors:** Christian Waydhas, Frank Hildebrand, Liping Liu

**Affiliations:** ^1^Department of Trauma, Hand and Reconstructive Surgery, Essen University Hospital, Essen, Germany; ^2^Department of Trauma and Reconstructive Surgery, University Hospital RWTH Aachen, Aachen, Germany; ^3^Emergency Intensive Care Unit, First Hospital of Lanzhou University, Lanzhou, Gansu, China

**Keywords:** severe trauma injury, trauma systems, prehospital trauma care, emergency room algorithms, intensive care treatments, first surgical phase, global surgery

## Introduction

Severe trauma is among the leading causes of death and morbidity in many age groups around the world. The chain of survival in critically ill injured patients starts on the scene, continues in the emergency department, and carries on in the first surgical phase and in the intensive care unit. The optimal care of such patients depends on both medical treatment and organizational management. Therefore, it is essential to examine both of these aspects if healthcare systems are to optimize the care of critically ill trauma patients in different parts of the world.

Provision of the best available medical care for severely injured patients might result in suboptimal outcomes if prehospital care, or any link in the path of the patients through the chain of survival, is not of similarly high quality. A close look at the various challenges faced by trauma systems—and their potential solutions—in different regions of the world will enable the identification of existing short-comings and may facilitate the exchange of relevant solutions and possible improvements to processes. Both unmet surgical needs and the cost effectiveness of surgical intervention should be addressed and potential solutions should be suggested, including those related to medical, training, and organizational issues.

In emergency and intensive care, many aspects of care are specific to trauma patients, and so general recommendations and guidelines may not cover the precise needs of injured patients in these settings. Furthermore, many treatment options are currently under scientific discussion. Therefore, new research results and a critical appraisal of the relevant up-to-date knowledge is provided to the readers herein.

## Non-technical skills and teamwork

A team of experts does not necessarily make an expert team. Starting from this observation, Alexandrino et al. reviewed some of the most important non-technical skills that are required not only in the emergency department, but also in the operating room, and they proposed ways to improve perioperative communication. Furthermore, they give a short appraisal of existing training courses.

## Interhospital transfer

The indication and decision of when to transfer a severely injured patient from one to another hospital are complex. Spering et al. described processes followed for the common, countrywide practice of the interhospital transfer of severely injured patients within a highly developed national network of trauma centers. In addition to their epidemiological overview, they were able to identify major factors that resulted in such a transfer. They also presented data indicating that the introduction of a trauma network resulted in an improved transfer policy.

## Anticoagulant and antithrombotic drugs

With an increasingly active aging population, greater numbers of injured patients requiring anticoagulant or antithrombotic treatment are presenting challenges to emergency and surgical teams. One study (Yamaji et al.) investigated the impacts of pre-trauma anticoagulant and antithrombotic treatment on mortality in patients with major trauma. They identified differences in outcomes between patients treated with anticoagulant vs. antithrombotic drugs. In this context, a registry study (Fitschen-Oestern et al.) focusing on an elderly population analyzed the effects of the application of tranexamic acid on outcomes in patients who were older than 50 years and receiving pre-existing treatment with anticoagulant or antithrombotic medication. In a third study (Schindler et al.), a high rate of intracranial bleeding was observed in elderly patients who were receiving anticoagulant or antithrombotic treatment before experiencing a low fall. As this observation was also made in asymptomatic patients, the findings hinted at the necessity of performing a head-CT scan in all such patients.

## Bleeding control and fracture management

Endovascular intervention techniques may significantly improve bleeding control and outcomes in severely injured patients. However, they require excellent infrastructure and organization, as delayed processes and other obstacles may diminish their potential benefits. Mizuno et al. analyzed the role of a time delay in transcatheter embolization in patients with pelvic fracture, and their findings substantiated the importance of fast intervention.

The Zürich group of Kalbas et al. presented a review and analysis of registry data that examined the best way to decide on the timing of surgical fracture stabilization in patients with multiple injuries. They discussed the values of different parameters and scores, and they propose the use of a decision tree.

## Ventilation and early mobilization

Ventilation and mobilization are central interventions during the intensive care treatment of severely injured patients. Although the general guidelines of ventilatory support do also apply to trauma patients, there may be specific conditions (e.g., pulmonary contusion, inhalation injury) that require more individualized treatment. Meregildo-Rodríguez et al. looked at the ventilation of patients with cervical spinal cord injury who may have specific requirements due to the lack of a primary lung injury and a lack of respiratory-muscle activity. The authors presented a systematic review with a special focus on tidal volumes.

The role and potential advantages of early mobilization in ventilated patients is still a Research Topic under discussion. Wang et al. present another meta-analysis, this time including only randomized controlled trials. They suggested taking a differentiated view with respect to effects on different outcome parameters.

## Prediction and quality indicators

Early identification of patients at increased risk of developing complications may help to enable stratification of patients into specific monitoring or treatment pathways, or for potential inclusion in studies trialing novel therapies. In a single-center study (Xu et al.), a predictive model for the “Persistent Inflammation, Immunosuppression, and Catabolism Syndrome” was developed and evaluated in trauma patients using parameters that are easily obtained. A nomogram for the calculation of risk was presented.

As has already been stressed in another manuscript on this Research Topic, traumatic brain injuries after low falls in the elderly population is of special interest and concern. It would, therefore, be helpful if specific predictors could be used to indicate the likely treatment courses, outcomes, and resource requirements of such patients. The research group of Forssten et al. identified five predictors of complications and mortality in elderly patients presenting with moderate (Glasgow Coma Scale 9–13) traumatic brain injury after ground-level falls through analyzing information from the database of the American College of Surgeons' Trauma Quality Improvement Program.

Mortality is the primary outcome measure in severely injured trauma victims, with excellent methods available for risk adjustment. However, quality indicators for survivors are rare, and most of those that exist lack validated tools for risk adjustments to improve comparability. Using data from the German Trauma Registry, Lefering et al. developed a model that predicts the length of stay of trauma-injury survivors; after using a dataset describing 108,175 patients, they validated the model in another dataset of more than 72,000 patients. They describe the prediction of patient requirements for ICU treatment (with a duration of more than 1 week) and length of ICU stay for these long-term patients. Their results suggest that this tool may be useful for future benchmarking.

The manuscripts presented on our Research Topic cover a wide range of contributions on subjects that appear to be of general interest for providers of care in the emergency departments, early surgical management, and intensive care treatment in addition to describing prediction models and indicators that may be used for quality control and benchmarking. We hope that readers will gain interesting and helpful information and insights for their own clinical practice and/or stimuli for their own research.

## Author contributions

CW: Writing – original draft, Writing – review & editing. FH: Writing – review & editing. LL: Writing – review & editing.

